# Conservation genetics of a rare Gerbil species: a comparison of the population genetic structures and demographic histories of the locally rare Pygmy Gerbil and the common Anderson's Gerbil

**DOI:** 10.1186/1472-6785-10-15

**Published:** 2010-06-02

**Authors:** Ron Rotkopf, Zvika Abramsky, Ofer Ovadia

**Affiliations:** 1 Department of Life Sciences, Ben-Gurion University of the Negev, Be'er-Sheva 84105, Israel

## Abstract

**Background:**

One of the major challenges in evolutionary biology is identifying rare species and devising management plans to protect them while also sustaining their genetic diversity. However, in attempting a broad understanding of rarity, single-species studies provide limited insights because they do not reveal whether the factors that affect rare species differ from those that affect more common species. To illustrate this important concept and to arrive at a better understanding of the form of rarity characterizing the rare *Gerbillus henleyi*, we explored its population genetic structure alongside that of the locally common *Gerbillus andersoni allenbyi*. We trapped gerbils in several locations in Israel's western and inner Negev sand dunes. We then extracted DNA from ear samples, and amplified two mitochondrial sequences: the control region (CR) and the *cytochrome oxidase 2 *gene (CO2).

**Results:**

Nucleotide diversity was low for all sequences, especially for the CR of *G. a. allenbyi*, which showed no diversity. We could not detect any significant population genetic structure in *G. henleyi*. In contrast, *G. a. allenbyi*'s CO2 sequence showed significant population genetic structure. Pairwise PhiPT comparisons showed low values for *G. henleyi *but high values for *G. a. allenbyi*. Analysis of the species' demographic history indicated that *G. henleyi*'s population size has not changed recently, and is under the influence of an ongoing bottleneck. The same analysis for *G. a. allenbyi *showed that this species has undergone a recent population expansion.

**Conclusions:**

Comparing the two species, the populations of *G. a. allenbyi *are more isolated from each other, likely due to the high habitat specificity characterizing this species. The bottleneck pattern found in *G. henleyi *may be the result of competition with larger gerbil species. This result, together with the broad habitat use and high turnover rate characterizing *G. henleyi*, may explain the low level of differentiation among its populations. The evidence for a recent population expansion of *G. a. allenbyi *fits well with known geomorphological data about the formation of the Negev sand dunes and paleontological data about this species' expansion throughout the Levant. In conclusion, we suggest that adopting a comparative approach as presented here can markedly improve our understanding of the causes and effects of rarity, which in turn can allow us to better protect biodiversity patterns.

## Background

Studying rare species and devising management plans to protect them constitute major themes in ecology [e.g. [1]]. Doing so allows us to preserve ecosystem integrity, and to prevent biodiversity loss. Classical views in evolutionary ecology define a rare species as one with a low abundance and/or a small range [2]. However, rarity can also be characterized by factors such as habitat specificity, taxonomic distinctness, and persistence through ecological or evolutionary time, all of which have been considered either as additional components by which rare species can be identified or as constraints which confer upon species with low abundances or small-sized ranges the rare status [2-4]. For instance, Rabinowitz [3] and Rabinowitz et al. [4] suggested categorizing plant species according to geographic range (large or small), local population size (large or small) and habitat specificity (wide or narrow). These three states generate eight possible combinations, seven of which constitute forms of rarity. A similar study carried out on mammals [5] revealed a bimodal pattern of rarity and commonness, with an overabundance of species in the relatively rarest and most common categories.

Identifying the causes of rarity often leads to the following question: Are rare species rare because they are only capable of exploiting a narrow range of environmental conditions, or because the spatial extent of the conditions they can exploit is highly restricted, or both? All species are limited to some extent by environmental variables (i.e. abiotic and biotic), but the question is whether there is a tendency for the abundances and range sizes of rare species to be predominantly constrained by particular factors or combinations of factors [2]. For instance, a species' rarity may indicate that it is being affected more heavily by competition or predation than a more common congener [e.g. [6]].

Recently, there has been a growing awareness among conservation biologists that molecular techniques can be better exploited to interpret the history, present status, and future prognosis of threatened species [7]. Moreover, combined with data from other disciplines (e.g. reproduction, infectious diseases, and field ecology), the synthesis offers valuable insight that is directly applicable to species management plans [7]. For example, the quest for the most suitable population for translocation or as a source for restoration could benefit immensely from recognizing locally adaptive genetic diversity within evolutionarily significant units. In such a case, adaptive genetic differences among populations can lead to outbreeding depression if divergent populations are mixed [8].

Attempts to devise a broad understanding of rarity will accrue only limited insight from single-species studies [9], which do not reveal how the factors that control abundances and range sizes vary between rare species and more common species [2]. As such, comparisons between rare species and common congeners are essential to gain further insight about rarity. For example, Wahlberg et al. [10] used data on an abundant butterfly species to estimate the parameters of the incidence function model of an endangered congener, thus demonstrating the usefulness of studying a rare species alongside a common congener. Yet, studies of this kind, especially on animals, are scarce [but see [11]].

To illustrate the importance of this comparative approach, we explored the population genetic structure of the rare Pygmy Gerbil (*Gerbillus henleyi*) and the locally common congener Anderson's Gerbil (*Gerbillus andersoni allenbyi*), while contrasting their demographic histories. Doing so enabled us to form a better mechanistic understanding of the type of rarity that characterizes the Pygmy Gerbil, i.e. rarity signified by widespread global distribution, low habitat specificity, and small population size. This form of rarity is especially difficult to explain, since one would expect such small populations to die out and that the species subsequently persist in fewer but larger populations.

Our working hypothesis was that the type of rarity evident in *G. henleyi *may persist in two forms that differ from one another in the level of connectivity between populations. Our null hypothesis was that the form of rarity exhibited by *G. henleyi *can persist by maintaining a high level of connectivity between populations. This hypothesis relies on two characteristics inherent to the species, its high mobility [12] and its ability to exploit several habitat types [12-15]. We thus predicted that since *G. henleyi *populations are not isolated, they should demonstrate fairly low levels of genetic differentiation. Alternatively, it is possible that *G. henleyi *exists in small, isolated populations. This latter pattern should be manifested by higher levels of genetic differentiation between populations.

**Figure 1:**
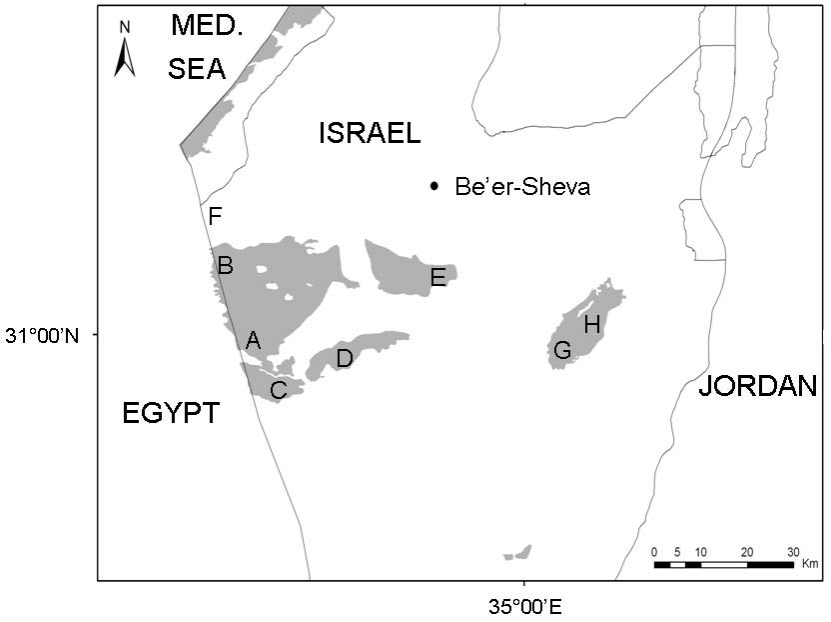
**Map of sampling locations**. A--Agur sands south (30°58'N, 34°24'E), B--Agur sands north (31°07'N, 34°20'E), C--Mifrasit (30°54'N, 34°27'E), D--Mashabim (30°58'N, 34°38'E), E--Nahal Secher (31°06'N, 34°48' E), F--Avshalom (31°12'N, 34°19'E), G--Mamshit (31°01'N, 35°04'E), H--Mishor Yamin (30°59'N, 35°04'E) and Mishor Rotem (31°03'N, 35°06'E). Sandy areas are marked in grey. Locations A-F are in the western Negev sands; locations G & H are in the inner sands.

Regarding *G. a. allenbyi*, we hypothesized that its larger body size (i.e. higher mobility) and larger population size (i.e. a potential source for dispersal events) will increase the connectivity between populations, especially those within the western Negev sand dunes. However, its high specificity to sand [16], relative to *G. henleyi *(see Methods), may decrease the connectivity between the western Negev sand dunes (Figure 1a-f) and the inner sands populations (Figure 1g, h) (no sandy connection exists between these two regions). We thus expected to find little genetic differentiation between populations in the western Negev sand dunes and high levels of genetic differentiation between populations of the western Negev sand dunes and the isolated populations of the inner Negev sands.

## Methods

### Study area

The four sand dune regions of Israel include: 1) the coastal and 2) western Negev dunes, which together form the eastern boundary of the Saharan sands and originate from material carried by the Nile and deposited along the Mediterranean coast, 3) the inland dunes of the Negev (Mishor Rotem, Mishor Yamin, and Mamshit), which derive from locally eroded tertiary rocks, and 4) the sands of the Arava valley, produced mostly by the erosion of local Nubian sandstones of the lower Cretaceous period [17]. *G. a. allenbyi *and *G. henleyi *both occur in the western and inland Negev dunes. No sandy connection exists between these two regions (Figure 1).

### Study species

The Pygmy Gerbil, *G. henleyi *(body mass 9.5 g), is a widely distributed species that ranges from the Arabian peninsula in the east to Morocco in the west and from the African Mediterranean coast in the north to Senegal, Burkina Faso, Chad, and Yemen in the south [12]. *G. henleyi *seems to be a generalist species-in Israel, it has been trapped in sand dunes, gravel plains, wadis, and on loess and cobble substrate adjacent to sand dunes [13-15], and it is considered rare on all those substrates [14,18]. In a study conducted in Makhtesh Ramon [12] average *G. henleyi *densities were between 0.9-2.3 individuals per hectare, depending on habitat type. The population of *G. henleyi *exhibits a high level of individual turnover, suggesting high mobility rates [12]. The average duration of uninterrupted presence of an individual on a sampling plot varies between different habitats, with permanent habitation found only in wadis and gravel plains [12]. *G. henleyi *is more mobile and its home ranges are less stable than those of either *G. dasyurus *[19] or *G. gerbillus *[12]. Negative interactions with congeners (specifically *G. a. allenbyi *and *G. pyramidum*) were found to be a major cause for the scarcity of *G. henleyi *in Israel [20].

Anderson's Gerbil, *G. a. allenbyi *(body mass 26 g), occurs along the Mediterranean coast from Tunisia through Libya and Egypt to the Sinai and Israel. In Israel, it occurs in sand dunes that are covered with vegetation, as well as on fossilized sandstone hills along the coastal plain from the Egyptian border and north to Mount Carmel, and in the western and northern Negev. Its distribution in Israel was originally reported to be limited to the coastal sand dunes [13]. Ritte et al. [21] found the species in the western Negev sand dunes, and later it was found in the inner sand dunes of Mishor Rotem [14]. On sand, population densities of *G. a. allenbyi *(40-120 individuals per hectare, according to Abramsky [22] are much higher than those of *G. henleyi *(1 individual per hectare, according to Shenbrot et al.[12]).

### Samples and laboratory procedures

We sampled *G*. *henleyi *and *G. a. allenbyi *populations at several locations in the western Negev and in the inner sands (Figure 1). In each location, our goal was to trap 20 individuals of each species by setting 320 Sherman folding live-traps (23 × 8 × 9 cm) for three consecutive nights. Baited with millet seeds, the traps were set in the late afternoon and checked at dawn. Sampling consisted of recording species, sex, head width, hind foot length, tail length, and body mass. We anesthetized each animal with isoflurane and collected a small sample of ear tissue in 95% ethanol. Animals that were trapped for a second time (identifiable by their clipped ears) were released without further sampling. All required permits for this work were obtained from Israel's National Parks Authority (NPA), permit no. 2006/26425.

We extracted total DNA from each ear sample using the DNEasy Tissue Kit (QIAGEN 2004) or the Wizard^® ^SV Genomic DNA Purification System (Promega). A fragment of the mitochondrial control region (335 bp) was amplified and sequenced using the CBT (5'-CCGCCATCAACACCCAAAGCTG-3') and MR1 (5'-CCCTGAAGTAAGAACCAG ATGCCTG-3') primers [23]. Another fragment, comprising part of the CO1 gene, the CO2 gene, and part of the ATPase 8 gene (1407 bp), was amplified and sequenced using the F6520 (5'-GCWGGMTTYGTNCACTGATTCCC-3') and R7927 (5'-GAGGMRAAWARATTTTCGTTCAT-3') primers [24]. Polymerase chain reaction (PCR) amplification was carried out using PCR-Ready™ High Specificity microtubes in a PTC-200 thermal cycler. Reactions were carried out in 25-μL volumes that included 1 μL of 10-μM primer and 2 μL of the DNA template. The cycle profile for the control region consisted of denaturation at 94°C (1 min), annealing at 63°C (1 min), and extension at 72°C (1 min) for 35 cycles. This profile was preceded by an initial cycle with a 5-min denaturation at 94°C, and it was followed by a final cycle with a 7-min extension step at 72°C. The only difference for the CO2 region was an annealing temperature of 53°C. PCR products were purified using the Invisorb^® ^Spin PCRapid kit and stored in TE at -20°C.

### Quantifying genetic diversity and population structure

We estimated genetic diversity at the mtDNA level by calculating the number of polymorphic sites, the number of haplotypes, and the nucleotide diversity, Pi (π - the average number of nucleotide differences per site between two sequences [25](equations 10.5 or 10.6). We tested for evidence of population structure using analysis of molecular variance (AMOVA), which was calculated with GenAlex, ver. 6.3 [26]. Additionally, levels of genetic divergence between samples were calculated with the fixation index PhiPT [27], an estimator that includes information on haplotype frequency and molecular distance (calculated with GenAlex, ver. 6.3). We tested for isolation by distance by comparing genetic against geographical distances using Mantel tests. The protein-coding sequence was divided into two separate sequences: partial CO1 and partial CO2. In each of these sequences, synonymous and non-synonymous substitutions were identified. Positions of substitutions are described as amino-acid locations inferred from an alignment with reference sequences of *Mus musculus *acquired from the NCBI RefSeq protein database [Genbank:YP001686700 for CO1, NP904331 for CO2].

Four haplotype networks (for *G. henleyi*'s CR, CO2 and the combination of both, and for *G. a. allenbyi*'s CO2) were constructed to examine the evolutionary relationships between mtDNA haplotypes, and thus identify geographically localized clades. The phylogenetic relationships between the mtDNA haplotypes were determined using the program NETWORK 4.5.1.6 [28]. Networks were calculated by the median-joining method (ε = 0) [29] and subsequent MP calculation [30].

### Inferring demographic histories

We used the D statistic of Tajima [31], D* and F* statistics of Fu and Li [32] and F_S _statistics of Fu [33] to test for deviation of sequence variation from evolutionary neutrality. Negative values of these statistics are most often attributed to positive selective sweeps (resulting in genetic hitchhiking), recent population growth, or background selection. Used in combination, these tests can provide evidence for or against particular evolutionary mechanisms [34]. All tests and calculations of significance from coalescent simulations were conducted using version 4.2 of DnaSP [35].

To test for traces of historic rapid population expansion, we used mismatch distribution analyses. We also created a segregating sites graph to examine the distribution of mutations throughout the population. The former distinguishes between the smooth, unimodal distribution of a recently expanded population shaped by the accumulation of mutations with minimal lineage loss and the "ragged", multimodal distributions shaped by mutations in equilibrium with stochastic lineage loss [36-38]. The significance of the "raggedness" statistic [39] was used to differentiate between the two patterns.

The use of mismatch distributions may generate spurious, but seemingly precise results. We thus also used a Bayesian coalescence framework to simulate the demographic history of both gerbil species. Specifically, we estimated the past population dynamics through time using the method of Bayesian skyline plot [40], implemented in BEAST [41]. This method allows estimating past population dynamics from a sample of molecular sequences without dependence on a pre-specified parametric model of demographic history. It takes into account both the error inherent in phylogenetic reconstruction and the stochastic error intrinsic to the coalescent process, and thus produces more correct estimates of statistical uncertainty. In addition, the Monte Carlo-Markov Chain (MCMC) sampling procedure, implemented in BEAST [41], allows simultaneous estimation of the ancestral genealogy, and parameters of the substitution process and demography. Sequences were simulated down the tree using the general time-reversible (GTR) model and empirical base frequencies, while also allowing for a heterogeneous (four-category gamma distribution) mutation rate among sites. The number of groups (*m*) was set to 20 for *G. henleyi*, and 5 for *G. a. allenbyi*. MCMC chains were run for 10,000,000 iterations of which the first 10% were discarded to allow for burn-in. Genealogies and model parameters were sampled every 1,000 iterations, and the results were summarized in piecewise-constant Bayesian skyline plots. We assumed a strict molecular clock model, where evolutionary rate was expressed in mutations per site per year, assuming it is normally distributed with a mean equal to 27.4 × 10^-9 ^[42], and a standard deviation of 10 × 10^-9^. We used a relatively high standard deviation due to the high variability in mutation fixation rates in different regions of the mitochondrion.

We also calculated the level of linkage disequilibrium (the proportion of polymorphic sites exhibiting significant, non-random associations). If a certain gene in the mitochondrion underwent strong positive selection, one would expect to find significant associations, because of genetic hitchhiking, between polymorphic sites. Slatkin [43] demonstrated that the probability of detecting linkage disequilibrium between (neutral) loci in mtDNA is greatly diminished in a population that recently expanded. This is a result of the accumulation of new mutations when haplotype loss is minimal. Only parsimony-informative sites were used in this analysis. All calculations were performed using DnaSP, version 4.2 [35].

### Phenotypic variability

If life history and morphology are entirely genetically determined, patterns of trait variation within and among different populations should be correlated with the degree of genetic differentiation they exhibit. If gene flow among different gerbil populations is restricted, then such morphological traits important to a species' interaction in an ecosystem may become geographically structured, so local populations will only contain a subset of the traits exhibited within a species [44], or be constrained in the extent to which they express certain traits. Exploring such geographically structured traits can reveal the extent to which local populations are non-interchangeable due to their distinct adaptations. To test if the morphological traits characterizing *G. henleyi *and *G. a. allenbyi *are geographically structured, we analyzed the log-transformed measurements of their hind foot length, tail length, and body mass using a multivariate analysis of variance (i.e. MANOVA) followed by univariate tests (i.e. ANOVAs), both conducted using SYSTAT v.11.

## Results

The results of our sampling efforts depended on the species and location sampled (Table 1). While *G. a. allenbyi *individuals were relatively easy to catch, specimens of *G. henleyi *were much harder to trap, which forced us to use smaller sample sizes. The same partial length (335 bp) of the control region (CR) was sequenced for all individuals. The CO2 sequence contains part of the CO1 gene, the CO2 gene, and part of the ATPase 8 gene. Because the entire sequence was too long to achieve high-quality readouts, we created a chimera sequence by joining the two ends which were readable. The lengths of the sequences eventually used were 1009 bp for *G. henleyi *and 927 bp for *G. a. allenbyi*.

**Table 1:** Number of gerbils caught at each sampling location

Location	*G. henleyi*	*G. a. allenbyi*
Agur South	9 (4/5)	20 (9/11)
Agur North	19 (7/12)	20 (9/11)
Mifrasit	19 (10/9)	20 (12/8)
Mashabim	13 (6/7)	20 (7/13)
Nachal Secher	---	5 (0/5)
Avshalom	---	20 (8/12)
Mishor Rotem	---	2 (0/2)
Mishor Yamin	---	20 (15/5)
Mamshit	4 (4/0)	14 (8/6)

In most locations, within three nights we easily exceeded the quota--dictated by the National Parks Authority as 20--for *G. a. allenbyi *individuals. This quota was not reached for *G. henleyi*, specimens of which were harder to catch. Even in areas where *G. henleyi *was present, some nights only one or two individuals were caught, and on other nights, no individuals were caught at all. Locations in the inner sands are indicated in italics, male/female numbers in parentheses.

No insertions or deletions were present in any of the sequences. There was a pronounced bias against G in both CR and CO2 fragments (mean G composition: 8.7% in *G. henleyi *CR, 12.8% in *G. henleyi *CO2, and 13.4% in *G. a. allenbyi *CO2). In both species, no stop codons were detected within the CO1 and CO2 sequences. All these results support the conclusion that the sequences represent mtDNA and are not nuclear pseudogenes. The CR sequence in *G. henleyi *(N = 63) contained 30 variable sites, 29 of which were parsimony-informative. A total of 16 haplotypes were identified, and nucleotide diversity (Pi) was 2.1%. The CO2 region in *G. henleyi *(N = 61) contained 52 polymorphic sites, 37 of which were parsimony-informative. A total of 25 haplotypes were identified, and nucleotide diversity was 0.8%. *G. henleyi*'s concatenated sequence of CR and CO2 (N = 60) contained 82 polymorphic sites, 66 of which were parsimony-informative. A total of 34 haplotypes were identified, and nucleotide diversity was 1.1%. The CR sequence in *G. a. allenbyi *(N = 141) contained no variable sites. The CO2 region in *G. a. allenbyi *(N = 69) contained 29 polymorphic sites, 21 of which were parsimony-informative. A total of 25 haplotypes were identified, and nucleotide diversity was 0.3%. These diversity levels are low for all the sequences, and as such, the number of polymorphic sites and nucleotide diversity per sampling location are correspondingly low (Table 2). In *G. henleyi*, the CO1 sequence contained nine synonymous substitutions. One non-synonymous substitution (in amino-acid 508) occurred in four individuals from the Agur sands. The CO2 sequence contained 23 synonymous substitutions. Five non-synonymous substitutions occurred in amino acids 54 (4 individuals from the western sands), 142 (2 individuals from the western sands), 182 (2 individuals from the western sands), 185 (2 individuals from the western sands), and 191 (3 individuals from the western sands). In *G. a. allenbyi*, the CO1 sequence contained six synonymous substitutions. Two non-synonymous substitutions occurred in amino acids 483 (16 individuals from the western sands) and 485 (2 individuals from the western sands). The CO2 sequence contained 12 synonymous substitutions. Three non-synonymous substitutions occurred in amino acids 8 (2 individuals from Mamshit in the inner sands), 21 (5 individuals from Mamshit), and 43 (2 individuals from the western sands).

**Table 2:** Number of polymorphic sites and nucleotide diversity per sampling location

	*G. henleyi*									*G. a. allenbyi*			
	**CR**			**CO2**			**CR+CO2**				**CO2**		**CR+CO2**

**Location**	**N**	**polymorphic sites**	**π**	**N**	**polymorphic sites**	**π**	**N**	**polymorphic sites**	**π**	**N**	**polymorphic sites**	**π**	**π**

Agur South	9	17	0.019	7	12	0.006	7	27	0.009	9	16	0.005	0.004
Agur North	19	28	0.025	19	38	0.008	19	66	0.012	10	9	0.004	0.003
Mifrasit	18	21	0.021	18	33	0.007	18	54	0.011	9	6	0.002	0.001
Mashabim	13	14	0.019	13	37	0.011	13	51	0.013	10	9	0.003	0.002
Nahal Secher	-									5	4	0.002	0.002
Avshalom	-									5	6	0.003	0.002
Mishor Yamin	-									12	2	0.001	<0.001
Mamshit	4	7	0.01	4	8	0.004	4	15	0.006	9	5	0.002	0.001

Diversity is low at all locations for both species. Inner sands locations are indicated in italics.

*G. a. allenbyi *had no polymorphic sites in the CR sequence.

AMOVA results from the analyses of the CR and CO2 sequences (individually and concatenated) in *G. henleyi *showed that genetic variation was solely attributed to within-population differences (100%) (Table 3). Moreover, the global PhiPT values were zero and non-significant, indicating no significant population structure. The analysis for the CO2 sequence in *G. a. allenbyi *(recall that there was no variation in the CR sequence of *G. a. allenbyi*, therefore concatenating both sequences would have had no effect on the results) showed that 87% of the genetic variation occurred within populations and 13% among populations. The high global PhiPT value (0.133, *p *= 0.001) indicates a significant population genetic structure. The very small (N ≤ 5) sample sizes from Nahal Secher, Avshalom, and Mishor Rotem led us to combine the samples from Nahal Secher and Mashabim, Avshalom and Agur North, and Mishor Rotem and Mishor Yamin for the AMOVA analysis. Grouping is a potential source of bias in such analyses. Note that Avshalom and Agur north are close to each other, as are Mishor Yamin and Mishor Rotem. Regarding Mashabim and Nahal Secher, they may seem further away from each other on the map than the other groupings, but geomorphologically and botanically, they are considered as locations within a single geographical unit [45]. Furthermore, conducting the analyses without individuals from these three smaller-sample locations did not have a qualitative effect on the results, suggesting that the grouping did not cause bias.

**Table 3:** AMOVA results for *G. henleyi *and *G. a. allenbyi *based on analyses of the CR and CO2 sequences

Species	Sequence	Source of variation	*df*	Percentage of variation	PhiPT
*G. henleyi*	CR	Within populations	58	100	0 (*P *= 0.435)
		Among populations	4	0	
	CO2	Within populations	56	100	0 (*P *= 0.463)
		Among populations	4	0	
	CR+CO2	Within populations	55	100	0 (*P *= 0.450)
		Among populations	4	0	
*G. a. allenbyi*	CO2	Within populations	61	87	0.133 (*P *= 0.001)
		Among populations	7	13	

All *G. henleyi *variation was explained by differences within populations, while 13% of the variation in *G. a. allenbyi*'s CO2 region was explained by differences among populations. *G. a. allenbyi*'s CR was not analyzed since there was no variance in these sequences.

Low values were obtained from pairwise PhiPT comparisons for the CR and CO2 concatenated sequence and for each sequence separately in *G. henleyi *(Table 4, see Additional file 1). The results for *G. a. allenbyi *(Table 5), on the other hand, contain several higher values (>0.2), indicating a great degree of isolation between populations [46]. The higher values come mostly from comparisons of the population in Mamshit with other populations, indicating that there is some isolation between the inner sands and the western Negev sand dunes. Mantel tests did not show significant correlations between genetic and geographical distances for any of the sequences (*G. a. allenbyi *CO2: *p *= 0.417; *G. henleyi *CR: *p *= 0.174, CO2: *p *= 0.381, CR+CO2: *p *= 0.649).

**Table 4:** Pairwise PhiPT values for *G. henleyi*'s combined CR & CO2 sequences

	Agur South	Agur North	Mifrasit	Mashabim	Mamshit
Agur South	0.000	0.396	0.300	0.252	0.291
Agur North	0.000	0.000	0.368	0.228	0.377
Mifrasit	0.006	0.000	0.000	0.363	0.204
Mashabim	0.027	0.016	0.005	0.000	0.415
*Mamshit*	0.000	0.000	0.065	0.000	0.000

The low values indicate low levels of differentiation between populations. Probability values based on 999 permutations are above the diagonal and PhiPT values are below the diagonal. Note that Mamshit (indicated in italics) is the only location from the inner sands.

**Table 5:** Pairwise PhiPT values for *G. a. allenbyi*'s CO2 sequence

	Agur South	Agur North	Mifrasit	Mashabim	Mishor Yamin	Mamshit
Agur South	0.000	0.433	0.012	0.103	0.049	0.003
Agur North	0.000	0.000	0.008	0.183	0.005	0.005
Mifrasit	0.103	0.175	0.000	0.010	0.001	0.001
Mashabim	0.059	0.030	0.200	0.000	0.011	0.004
*Mishor Yamin*	0.077	0.167	0.189	0.209	0.000	0.002
*Mamshit*	0.126	0.201	0.226	0.239	0.219	0.000

Probability values based on 999 permutations are above the diagonal and PhiPT values are below the diagonal. Higher values (≥0.2) show that populations of this species exhibit a great degree of differentiation, especially between populations from Mamshit and from the western sands. Inner sands locations are indicated in italics.

When examining the haplotype networks (see Additional file 2), the *G. a. allenbyi *network showed noticeable haplotype clustering of the CO2 sequences from the inner sands region. Haplotypes unique to the inner sands are closely related to a common haplotype which is shared by individuals from both regions. In the *G. henleyi *CR network, no haplotype is unique to the inner sands, and haplotypes which are found in the inner sands are not clustered together. In the *G. henleyi *CO2 and concatenated sequence networks, one haplotype is unique to the inner sands, showing only a weak connection to the other inner sands haplotypes.

**Figure 2:**
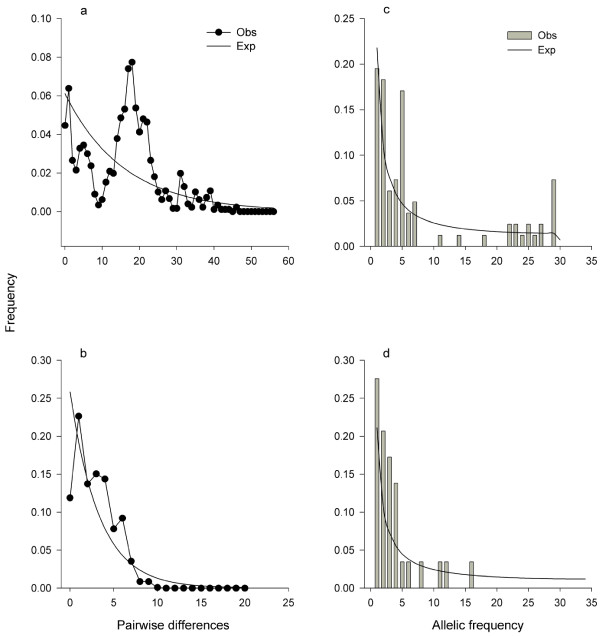
**Pairwise differences and allelic frequency graphs for *G. henleyi*'s combined CR and CO2 (a, c), and for *G. a. allenbyi*'s CO2 (b, d)**. The ragged pattern in the pairwise differences graph in *G. henleyi *indicates that this species has maintained a stable population size over a long period of time. The wave-like pattern in the *G. a. allenbyi *graph, however, indicates that this species underwent a recent population expansion. The allelic frequency graphs indicate that older mutations (mutations that are shared by many individuals) predominate in *G. henleyi *and, consistent with a recent population expansion, relatively new mutations predominate in *G. a. allenbyi*.

Neutrality tests resulted in positive values for *G. henleyi*'s CR, non-significant (negative) values for *G. henleyi*'s CO2 and for the concatenated sequence, and negative values for *G. a. allenbyi*'s CO2 sequence (Table 6). Negative values of these indices indicate a recent population expansion, while positive values indicate some other demographic force at work, such as a recent or historical bottleneck. The mismatch distribution analysis for *G. henleyi*'s CR and CO2 combined sequence (Figure 2a) shows a ragged distribution (r = 0.005, *p *= N.S.), indicative of a population which has been stable in size for a long period of time. The analysis of *G. a. allenbyi*'s CO2 (Figure 2b) shows a wave-like distribution (r = 0.028, *p *= 0.056), possibly indicative of a recent population expansion. The peaks in the graph are a result of the analysis being conducted on the entire dataset, which includes populations which are highly differentiated. A smoother pattern is evident when the western Negev populations are analyzed separately. The segregating sites analyses (Figure 2c, d) both show an excess of new mutations compared to the amount expected by a neutral model. *G. henleyi*'s analysis shows an excess of older mutations (mutations shared by many individuals; see Additional file 3).

**Table 6:** Neutrality tests

Species	Sequence	D	Fs	D*	F*
*G. henleyi*	CR	0.33	0.54	1.58†	1.34*
	CO2	-0.88	-3.76	-1.22	-1.3
	CR+CO2	-0.58	-4.95	-0.03	-0.29
*G. a. allenbyi*	CO2	-1.66†	-15.26‡	-0.57	-1.17

D, Tajima's statistic (Tajima 1989); Fs, Fu's statistic (Fu 1997); D* and F*, Fu and Li's statistics (Fu & Li 1993). Values are non-significant unless indicated otherwise. * P = 0.06; † P < 0.02; ‡ P < 0.001. Significance was determined using coalescent simulations in DnaSP ver. 4.2.

The approximate time since expansion can be estimated by the formula *t = τ/2u *[37]; *t *= time in years, *τ *= peak number of mismatches accumulated in mutational time since expansion where this time is measured in units of generations divided by 2*u*, and *u *= mutation rate per nucleotide site per generation multiplied by the number of nucleotides compared (in this case 1262 bp for *G. a. allenbyi*). *τ *was estimated at 1.59 from the mismatch distributions. This translates to an initiation of expansion of *G. a. allenbyi *about 23000-32000 years BP if we assume a generation interval of one year (*G. a. allenbyi*'s survivorship in nature is usually one year, during which it reproduces once) and a substitution rate of 19.4-27.4 × 10^-9 ^per nucleotide site, and year as previously estimated for the control region and protein-coding regions of mammalian mtDNA [42]. This date should be regarded as preliminary owing to the small size of the analyzed sequence and rough estimation of the gene's mutation rate.

**Figure 3:**
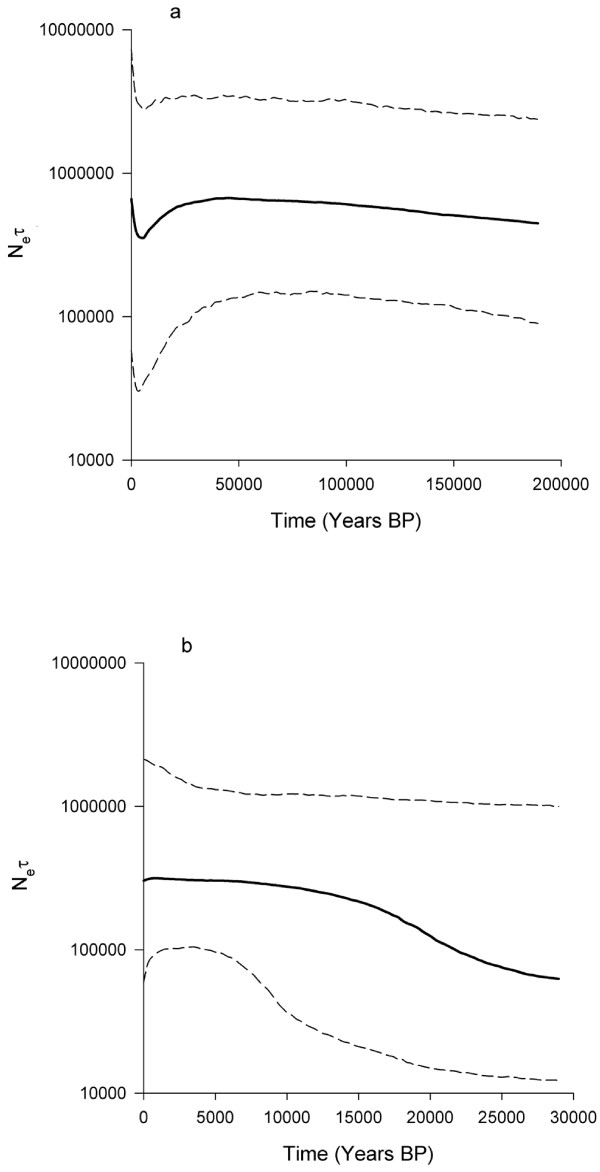
**A Bayesian skyline plot derived from an alignment of concatenated CR+CO2 sequences of *G. henleyi *(a) and *G. a. allenbyi *(b)**. The x-axis is in units of years before present, and the y-axis is equal to N_e_τ (the product of the effective population size and the generation length in years). The thick solid line is the median estimate, and the dashed lines show the 95% highest posterior density (HPD) interval. Sequences were simulated down the tree using the general time-reversible (GTR) model and empirical base frequencies, while also allowing for a heterogeneous (four-category gamma distribution) mutation rate among sites. The number of groups (*m*) was set to 20 for *G. henleyi*, and 5 for *G. a. allenbyi*. MCMC chains were run for 10,000,000 iterations of which the first 10% were discarded to allow for burn-in. Genealogies and model parameters were sampled every 1,000 iterations. Utilizing a strict molecular clock model, evolutionary rate was expressed in mutations per site per year, assuming it is normally distributed with a mean equal to 27.4 × 10^-9 ^[42], and a standard deviation of 10 × 10^-9^.

To investigate the behaviour of the Bayesian skyline plot model, we analyzed the sequence data of both species using BEAST. Sequences were simulated down the tree using the general time-reversible (GTR) model, while also allowing for a heterogeneous (four-category gamma distribution) mutation rate among sites. The number of groups (*m*) was set to 20 for *G. henleyi*, and 5 for *G. a. allenbyi*. The results are summarized in a Bayesian skyline plot (Figure 3). The *G. henleyi *model shows a steady increase in population size over ~150,000 years, followed by a sudden decrease, reaching a minimal population size ~6000 BP. These results are consistent with the mismatch distribution analysis, implying a bottleneck for *G. henleyi*. The *G. a. allenbyi *model shows an increase of ~500% in population size between 10,000-30,000 BP, further strengthening the conclusion that this species has undergone a recent population expansion.

The proportion of polymorphic sites exhibiting a significant, non-random association was 117/406 (28.82%) for *G. henleyi *CR, 169/528 (32.01%) for *G. henleyi *CO2, 501/1891 (26.49%) for *G. henleyi *concatenated sequence and 26/210 (12.38%) for *G. a. allenbyi *CO2 [After Bonferroni correction: 53/406 (13.05%), 80/528 (15.15%), 175/1891 (9.25%) and 6/210 (2.86%), respectively]. These relatively low values indicate no significant association between different mutations in the examined sequences.

No significant differences in body size were detected between male and female *G. henleyi *(MANOVA, Wilks' Lambda = 0.884; *F*_3,40 _= 1.755; *p *= 0.171; Figure 3a, b, c). Population-of-origin also had no significant effect on body size (MANOVA: Wilks' Lambda = 0.696; *F*_12,106 _= 1.300; *p *= 0.229), but there were significant differences (*F*_4,42 _= 3.130; *p *= 0.024) in body mass between populations. No significant differences were found between regions, i.e. western vs. inner sands (MANOVA: Wilks' Lambda = 0.989; *F*_3,48 _= 0.178; *p *= 0.911). The interaction between population and sex was not significant (MANOVA: Wilks' Lambda = 0.795; *F*_12,106 _= 0.799; *p *= 0.651).

**Figure 4:**
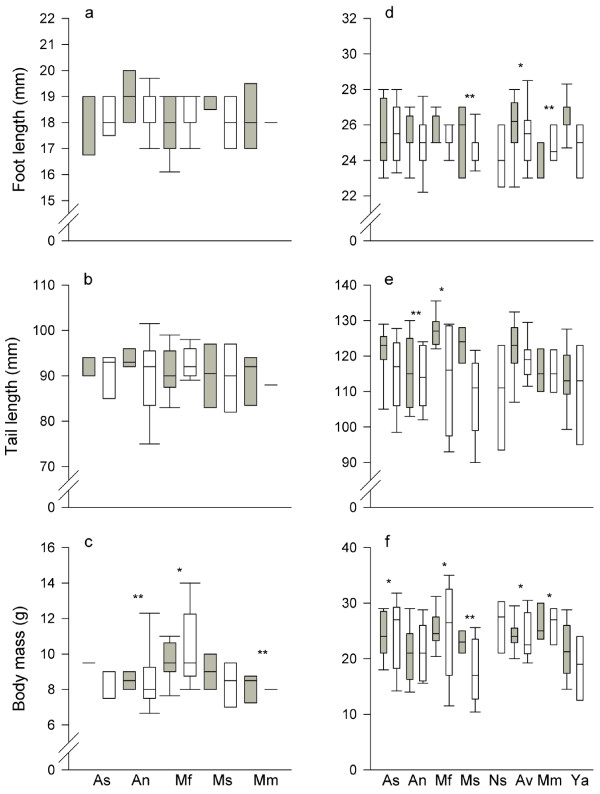
**Hind foot length, tail length and body mass for *G. henleyi *(a,b,c) and *G. a. allenbyi *(d,e,f)**. As-Agur south; An-Agur north; Mf-Mifrasit; Ms-Mashabim; Ns-Nahal Secher; Av-Avshalom; Mm-Mamshit; Ya-Mishor Yamin. Gray boxes indicate males, white boxes indicate females. Statistical analysis was performed on log-transformed data. Populations marked by an asterisk showed significant differences from populations marked by a double asterisk. Note that only females were caught in Nahal Secher.

Because only female *G. a. allenbyi *individuals were caught in Nahal Secher (Figure 4d, e, f), its sample was not used for the analysis. MANOVA found significant (Wilks' Lambda = 0.888; *F*_3,117 _= 4.938; *p *= 0.003) differences between the sexes. Sex had a significant effect (*F*_1,119 _= 6.730; *p *= 0.011) on tail length (males have longer tails than females) but not on foot length (*F*_1,119 _= 1.335; *p *= 0.250) or body mass (*F*_1,119 _= 0.031; *p *= 0.861). There were significant differences in body size between populations (MANOVA: Wilks' Lambda = 0.606, *F*_18,331 _= 3.572, *p *< 0.001; Univariate tests: *F*_6,119 _= 4.109, *p *= 0.001 for tail length, *F*_6,119 _= 5.535, *p *< 0.001 for body mass, and *F*_6,119 _= 2.082, *p *= 0.060 for foot length). Individuals from the western Negev were found to be larger (Wilks' Lambda = 0.931; *F*_3,129 _= 3.192; *p *= 0.026). No significant interaction was found between sex and population-of-origin (Wilks' Lambda = 0.832; *F*_18,331 _= 1.236; *p *= 0.230).

## Discussion

The purpose of this research was to demonstrate the importance of adopting a comparative approach in population and conservation genetic studies. To elucidate the links between the genetic make-up, phenotypic variation, and type of rarity characterizing *G. henleyi*, we compared its population genetic structure with that of *G. a. allenbyi*. Specifically, we compared the genetic structures and demographic histories of common and rare species in their continuous ranges and in isolated populations, while investigating a possible association between rarity and increased genetic differentiation.

Previous research has indicated that *G. henleyi *is considered rare wherever it is found [14,18]. In our study, *G. henleyi *was indeed found to be the rarer species-it occurred in lower numbers than *G. a. allenbyi*, and the sampling effort required to collect reasonable sample sizes was much higher. *Gerbillus henleyi*'s rarity is signified by widespread global distribution, low habitat specificity, and small population size. Examining worldwide rarity of mammals, Yu and Dobson [5] showed that only 3/255 of the rodents they examined (1.17% and 4.78% of mammals across all orders) possessed this type of rarity.

The level of genetic diversity, for all samples, was generally low for both species and for both sequences, culminating in the CR of *G. a. allenbyi*, which showed no variation whatsoever. This result was unexpected, since CR sequences are normally the most variable regions in mtDNA [47]. For example, a study examining the entire CR of red-backed voles [48] found that between 12% and 40% of the nucleotide positions were variable, depending on their locations within the CR. However, results such as ours are not unheard of-a study of introduced *Rattus rattus *in Madagascar [49] found 2.6% variable sites, a value which is also considered very low, in an analysis done on part of the control region. Furthermore, in a recent study examining mutation rates throughout the mtDNA of mice [50], a very low mutation rate was observed for the CR. A mechanism to explain this observation remains elusive.

The source of all the molecular variance in *G. henleyi *was found to be differences within rather than among populations. All pairwise comparisons produced low PhiPT values for analyses of the CR and CO2 sequences. In conclusion, the genetic structure analysis suggested that *G. henleyi *populations are not isolated, thus supporting the hypothesis that this species is evenly distributed throughout its geographical range, possibly owing both to its ability to utilize several habitat types and to its high mobility [12]. The lack of differentiation between the inner sands and western Negev populations of *G. henleyi *may indicate that this species has adopted a nomadic strategy to compensate for its small population size [e.g. [51]]. However, this explanation should be more thoroughly examined, because such small animals are usually not known to travel such long distances as those that exist between the inner sands and western Negev sands. In addition, it does not explain why the overall genetic diversity is lower than expected for a neutral population.

In the CO2 region of *G. a. allenbyi*, differences among populations accounted for 13% of the molecular variance, while 87% of the variance was accounted for by differences within populations (since no variation was found in the CR sequence of *G. a. allenbyi*, the exact same results were obtained when analyzing the concatenated sequence). PhiPT values for this species were much higher than for *G. henleyi*, and the highest values were obtained from comparisons of inner sands populations (especially Mamshit) with those of the western Negev, and also from the comparison of Mamshit and Mishor Yamin, both of which are located in the inner sands. The western Negev populations show relatively little to moderate levels of isolation from each other. However, the comparison of the Mifrasit and Mashabim populations gives a high PhiPT value of 0.2, indicating the potential for a high degree of isolation between populations even within the western sands. Other noteworthy moderate PhiPT values are those for Mishor Yamin and Mamshit vs. Agur South. Mishor Yamin and Mamshit are both part of the inner sands (thus rendering the relatively high degree of isolation between them surprising), and no sandy connection exists between these sands and the dunes of the western Negev more than 40 km west. Therefore, comparisons of the Mamshit and western Negev populations produce high PhiPT values. Why comparisons of Agur South with inner sands populations should be different in this respect remains unclear. One possibility is that individual *G. a. allenbyi *were transported by humans between Agur South and the inner sands at some point in the last few decades, a scenario that is supported by a study from 1957 [13], which found no *G. a. allenbyi *in the inner sands, and by the first announcement of their presence in Mishor Rotem only three decades later [14]. If this species only recently arrived to the inner sands, then it is indeed possible that these populations still resemble their source population even though they are, in fact, isolated. This explanation, however, is problematic because the degree of mtDNA divergence found between the Mamshit and the western Negev populations is not likely to have arisen over such a short period of time.

Three theoretical scenarios offer explanations for exceedingly low levels of genetic diversity and differentiation: a) selective sweep - a new mutation spreads out through positive selection, other genes hitchhike; should lead to strong linkage between polymorphic sites; b) background selection - purifying selection on a certain gene weeds out genetic variants, a process that is recognizable by the low accumulation of neutral mutations and low linkage disequilibrium (For example, this was the case with the protein-coding sequence CO2); and c) founder effect, or a rapid population expansion after a bottleneck, is characterized by low linkage disequilibrium. All three of these scenarios may help explain low genetic differentiation even without gene flow between the populations. Only the first and second scenarios are mutually exclusive.

In *G. henleyi*, two of the CR neutrality indices were positive, indicating an excess of older mutations, i.e. the entire population developed from a limited number of ancestors, but that population is still in a bottleneck (Note that this pattern was not evident when analyzing only the CO2 region, nor when analyzing the concatenated sequence). All the indices were negative for *G. a. allenbyi*, suggesting that this species underwent a recent population expansion. The low values detected by linkage disequilibrium tests for both species rule out the possibility of a selective sweep in either case. Both the ragged pattern produced by the pairwise-distribution analysis of *G. henleyi *and the Bayesian skyline plot indicate that its small population has not grown in many years. Similarly, the analysis of segregating sites indicated the existence of numerous old mutations that imply the presence of a limited number of common ancestors, which together attest to a more genetically variable past and suggest that the population experienced a bottleneck at some point. The experimental pattern of *G. a. allenbyi *resembled the wave-like pattern expected for a population that has undergone a historic change in population size. The segregating sites analysis revealed multiple new mutations-a pattern also indicative of a recent population expansion (which initiated at ~23,000-32,000 BP). Furthermore, the *G. a. allenbyi *Bayesian skyline plot showed an increase of ~500% in population size between 10,000-30,000 BP, further strengthening the conclusion that this species has undergone a recent population expansion. Aeolian sand became geologically significant in the western Negev around 25,000-30,000 BP. Sand dunes in considerable amounts are dated to 18,000-10,000 BP [45,52] and this agrees with the paleontological data used to evaluate this species' expansion in the Levant [53]. We interpret this to mean that the expansion of *G. a. allenbyi *coincided with the incursion of sands from the Nile Delta during the upper quaternary. Our expansion calculation should be regarded as preliminary due to the small size of the analyzed sequence and rough estimation of the gene's mutation rate.

Our analyses revealed some unequivocal differences between *G. henleyi *and *G. a. allenbyi*. Existing in small populations characterized by low levels of genetic diversity and differentiation, *G. henleyi *appears to be in the midst of a population bottleneck. Although the reasons for this bottleneck are not clear, a possible explanation may rest on this species' inferior ability to compete with more common and stronger gerbil species such as *G. pyramidum *and *G. a. allenbyi *[20]. The rare status of *G. henleyi *may affect its overall genetic diversity, but it does not seem to be affecting the amount of genetic differentiation between its populations. *G. a. allenbyi *is a larger, stronger, more common species, and the reasons for its low genetic diversity, especially in the control region, remain unclear. Populations of *G. a. allenbyi *were shown to be more isolated than those of *G. henleyi*, the most feasible explanation of which could be *G. a. allenbyi*'s higher habitat specificity.

Our genetic analyses are based on mtDNA data, which is haploid and maternally inherited. This is a widely used marker, although it may suffer from bias due to sex-specific dispersal patterns. Clearly, further research should be done in order to explore the extent to which patterns which arise from the mtDNA analysis conform to those arising from other genetic markers screening the nuclear genome as well, such as microsatellites or AFLP.

To test if different gerbil populations exhibit phenotypic divergence, we conducted morphometric analyses. Exploring geographically structured traits can reveal the extent to which local populations are non-interchangeable due to their distinct adaptations. No sexual size dimorphism was found in *G. henleyi*, but body mass varied significantly among populations. Regarding *G. a. allenbyi*, males were larger and had longer tails than females. This agrees with previous work on this species [22,54] which found that the mean body mass of males is higher than that of females. There were also clear phenotypic variations-e.g. foot length, tail length, and body mass differed significantly among *G. a. allenbyi *populations, and in a comparison between regions, individuals from the western sands were found to be larger than individuals from the inner sands. In a previous study [54], location also significantly affected body mass. However, body mass may be a problematic variable from which to infer genetic differences between populations-animals collected by Wahrman and Gourevitz [55] showed no significant differences in body mass between locations, after exposure to laboratory conditions for 2-4 weeks. Thus, it may be concluded that the differences in body mass between locations probably reflect a response to different environmental conditions, and have no or little genetic basis [54].

## Conclusions

Our findings indicate that *G. henleyi*'s type of rarity may be explained by two factors that are not mutually exclusive-high mobility rates, which decrease the level of divergence between populations, and an ongoing bottleneck, which lowers both the overall and within-population genetic diversity. It is important to note that the high mobility rates of *G. henleyi *are inferred from its high level of individual turnover [12]; we do not have direct data on this species' ability to traverse long distances. However, it seems unreasonable to assume that an isolated population of so few individuals can survive for such a long time devoid of contact with other populations of conspecifics. The comparison between *G. a. allenbyi *and *G. henleyi *surprisingly revealed that the more common species exhibited higher levels of divergence between populations than the rare species. This may indicate that habitat specificity (which is stronger in *G. a. allenbyi*) plays a more important role than the degree of rarity in determining a species' genetic variation. We suggest that adopting a comparative approach as presented here could largely improve our understanding of the causes and effects of rarity. Such an understanding should enable us to better protect ecosystem integrity and prevent biodiversity loss.

## Authors' contributions

RR carried out the field work and the molecular genetic studies, and drafted the manuscript. ZA instructed RR in carrying out the field work and participated in the study's design. OO participated in the study's design, instructed RR in carrying out the entire research and performing the genetic and statistical analyses, and contributed towards drafting the manuscript. All authors read and approved the final manuscript.

## Supplementary Material

Additional file 1 **Pairwise PhiPT values for *G. henleyi*'s CR & CO2 sequences**. The low values indicate low levels of differentiation between populations. Probability values based on 999 permutations are above the diagonal and PhiPT values are below the diagonal. Note that Mamshit (indicated in italics) is the only location from the inner sands. Click here for file

Additional file 2 **Haplotype networks of the four sequences (*G. a. allenbyi *CO2, *G. henleyi *CO2, CR and concatenated sequence)**. The networks describe the distances between haplotypes for the three sequences. Circle sizes are proportional to the number of individuals with a certain haplotype. Yellow areas represent individuals from the western Negev sands; brown areas represent individuals from the inner sands. Red circles represent theoretical intermediate haplotypes. The number of mutations is represented by ticks on the lines; a clean line indicates one mutation. In the *G. a. allenbyi *network, there appear haplotypes which are unique to the inner sands, and are clustered close to a common haplotype which is shared by both regions. In the *G. henleyi *CR network, there appear no haplotypes which are unique to the inner sands, and no clustering is evident. In the CO2 and concatenated sequence network, one haplotype is unique to the inner sands, but it shows no clear connection to the other inner sands haplotypes. Click here for file

Additional file 3 **Pairwise differences and allelic frequency graphs for *G. henleyi*'s CR (a, c) and CO2 (b, d)**. The ragged pattern in the pairwise differences graphs for both sequences in *G. henleyi *indicates that this species has maintained a stable population size over a long period of time. The allelic frequency graphs indicate that older mutations (mutations that are shared by many individuals) predominate in *G. henleyi*, suggesting that this species has experienced a bottleneck. Click here for file

## References

[B1] Meffe GK Carroll CR Principles of Conservation Biology 1997 Sunderland, MA: Sinauer Associates

[B2] Gaston KJ Rarity 1994 London, UK: Chapman & Hall

[B3] Rabinowitz D Synge H Seven forms of rarity The Biological Aspects of Rare Plant Conservation 1981 New York: John Wiley & Sons 205 217

[B4] Rabinowitz D Cairns S Dillon T Soulé ME Seven forms of rarity and their frequency in the flora of the British Isles Conservation Biology: the Science of Scarcity and Diversity 1986 Sunderland, MA: Sinauer Associates 182 204

[B5] Yu JP Dobson FS Seven forms of rarity in mammals Journal of Biogeography 2000 27 131 139 10.1046/j.1365-2699.2000.00366.x

[B6] Krystufek B Buzan EV Rarity and decline in palaeoendemic Martino's vole *Dinaromys bogdanovi* Mammal Review 2008 38 267 284 10.1111/j.1365-2907.2008.00127.x

[B7] O'Brien SJ A Role for Molecular-Genetics in Biological Conservation Proceedings of the National Academy of Sciences of the United States of America 1994 91 5748 5755 10.1073/pnas.91.13.5748 7912434 PMC44074

[B8] Storfer A Gene flow and endangered species translocations: a topic revisited Biological Conservation 1999 87 173 180 10.1016/S0006-3207(98)00066-4

[B9] Lindenmayer DB Fischer J Felton A Montague-Drake R Manning AD Simberloff D Youngentob K Saunders D Wilson D Felton AM The complementarity of single-species and ecosystem-oriented research in conservation research Oikos 2007 116 1220 1226 10.1111/j.0030-1299.2007.15683.x

[B10] Wahlberg N Moilanen A Hanski I Predicting the occurrence of endangered species in fragmented landscapes Science 1996 273 1536 1538 10.1126/science.273.5281.1536

[B11] Akst EP Boersma PD Fleischer RC A comparison of genetic diversity between the Galapagos Penguin and the Magellanic Penguin Conservation Genetics 2002 3 375 383 10.1023/A:1020555303124

[B12] Shenbrot G Krasnov B Khokhlova I On the Biology of Gerbillus-Henleyi (Rodentia, Gerbillidae) in the Negev Highlands, Israel Mammalia 1994 58 581 589 10.1515/mamm.1994.58.4.581

[B13] Zahavi A Wahrman J The cytotaxonomy, ecology and evolution of the gerbils and jirds of Israel (Rodentia: Gerbillinae) Mammalia 1957 21 341 380 10.1515/mamm.1957.21.4.341

[B14] Abramsky Z Brand S Rosenzweig M Geographical Ecology of Gerbilline Rodents in Sand Dune Habitats of Israel Journal of Biogeography 1985 12 363 372 10.2307/2844867

[B15] Mendelssohn H Yom-Tov Y Alon A Mammals Plants and Animals of the Land of Israel: An Illustrated Encyclopedia 1987 7 Tel-Aviv: Ministry of Defense Publishing House & The Society for the Protection of Nature in Israel 170 174

[B16] Abramsky Z Rosenzweig ML Pinshow B Brown JS Kotler B Mitchell WA Habitat Selection - an Experimental Field-Test with 2 Gerbil Species Ecology 1990 71 2358 2369 10.2307/1938646

[B17] Yom-Tov Y Yom-Tov Y, Tchernov E The zoogeography of the birds and mammals of Israel The zoogeography of Israel 1988 Dordrecht, The Netherlands: Dr. W. Junk Publishers 389 410

[B18] Mendelssohn H Yom-Tov Y Mammalia of Israel 1999 Jerusalem: The Israel Academy of Sciences and Humanities

[B19] Khokhlova I Krasnov B Shenbrot G Degen A Factors determining patterns of seasonal body mass changes in several rodent species in the Ramon erosion cirque, Negev highlands, Israel Zoologicheskii Zh 1994 73 115 121

[B20] Abramsky Z Rosenzweig ML Elbaz M Ziv Y Does interspecific competition from congeners cause the scarcity of Gerbillus henleyi in productive sandy desert habitats? Journal of Animal Ecology 2005 74 567 578

[B21] Ritte U Haim A Neufeld E Use of Electrophoretic Patterns of Hemoglobin for Identification of Israeli Gerbils (Genus Gerbillus-Rodentia-Gerbillinae) Israel Journal of Zoology 1976 25 52 60

[B22] Abramsky Z Population Biology of Gerbillus-Allenbyi in Northern Israel Mammalia 1984 48 197 206 10.1515/mamm.1984.48.2.197

[B23] Morzunov SP Rowe JE Ksiazek TG Peters CJ StJeor SC Nichol ST Genetic analysis of the diversity and origin of hantaviruses in Peromyscus leucopus mice in North America Journal of Virology 1998 72 57 64 9420200 10.1128/jvi.72.1.57-64.1998 PMC109349

[B24] Steppan SJ Adkins RM Spinks PQ Hale C Multigene phylogeny of the Old World mice, Murinae, reveals distinct geographic lineages and the declining utility of mitochondrial genes compared to nuclear genes Molecular Phylogenetics and Evolution 2005 37 370 388 10.1016/j.ympev.2005.04.016 15975830

[B25] Nei M Molecular Evolutionary Genetics 1987 New York: Columbia University Press

[B26] Peakall R Smouse PE GENALEX 6: genetic analysis in Excel. Population genetic software for teaching and research Molecular Ecology Notes 2006 6 288 295 10.1111/j.1471-8286.2005.01155.x PMC3463245 22820204

[B27] Excoffier L Smouse PE Quattro JM Analysis of Molecular Variance Inferred from Metric Distances among DNA Haplotypes - Application to Human Mitochondrial-DNA Restriction Data Genetics 1992 131 479 491 1644282 10.1093/genetics/131.2.479 PMC1205020

[B28] Fluxus Engineering http://www.fluxus-engineering.com

[B29] Bandelt HJ Forster P Sykes BC Richards MB Mitochondrial Portraits of Human-Populations Using Median Networks Genetics 1995 141 743 753 8647407 10.1093/genetics/141.2.743 PMC1206770

[B30] Polzin T Daneshmand SV On Steiner trees and minimum spanning trees in hypergraphs Operations Research Letters 2003 31 12 20 10.1016/S0167-6377(02)00185-2

[B31] Tajima F The Effect of Change in Population-Size on DNA Polymorphism Genetics 1989 123 597 601 2599369 10.1093/genetics/123.3.597 PMC1203832

[B32] Fu YX Li WH Statistical Tests of Neutrality of Mutations Genetics 1993 133 693 709 8454210 10.1093/genetics/133.3.693 PMC1205353

[B33] Fu YX Statistical tests of neutrality of mutations against population growth, hitchhiking and background selection Genetics 1997 147 915 925 9335623 10.1093/genetics/147.2.915 PMC1208208

[B34] Venkatesan M Westbrook CJ Hauer MC Rasgon JL Evidence for a population expansion in the West Nile Virus vector Culex tarsalis Molecular Biology and Evolution 2007 24 1208 1218 10.1093/molbev/msm040 17339636 PMC4299762

[B35] Rozas J Sanchez-DelBarrio JC Messeguer X Rozas R DnaSP, DNA polymorphism analyses by the coalescent and other methods Bioinformatics 2003 19 2496 2497 10.1093/bioinformatics/btg359 14668244

[B36] Slatkin M Hudson RR Pairwise Comparisons of Mitochondrial-DNA Sequences in Stable and Exponentially Growing Populations Genetics 1991 129 555 562 1743491 10.1093/genetics/129.2.555 PMC1204643

[B37] Rogers AR Harpending H Population-Growth Makes Waves in the Distribution of Pairwise Genetic-Differences Molecular Biology and Evolution 1992 9 552 569 1316531 10.1093/oxfordjournals.molbev.a040727

[B38] Rogers AR Genetic-Evidence for a Pleistocene Population Explosion Evolution 1995 49 608 615 10.2307/2410314 28565146

[B39] Harpending HC Sherry ST Rogers AR Stoneking M The Genetic-Structure of Ancient Human-Populations Current Anthropology 1993 34 483 496 10.1086/204195

[B40] Drummond AJ Rambaut A Shapiro B Pybus OG Bayesian coalescent inference of past population dynamics from molecular sequences Molecular Biology and Evolution 2005 22 1185 1192 10.1093/molbev/msi103 15703244

[B41] Drummond AJ Rambaut A BEAST: Bayesian evolutionary analysis by sampling trees Bmc Evolutionary Biology 2007 7 10.1186/1471-2148-7-214 17996036 PMC2247476

[B42] Pesole G Gissi C De Chirico A Saccone C Nucleotide substitution rate of mammalian mitochondrial genomes Journal of Molecular Evolution 1999 48 427 434 10.1007/PL00006487 10079281

[B43] Slatkin M Linkage Disequilibrium in Growing and Stable-Populations Genetics 1994 137 331 336 8056320 10.1093/genetics/137.1.331 PMC1205949

[B44] Althoff DM Thompson JN Geographic structure in the searching behaviour of a specialist parasitoid: combining molecular and behavioural approaches Journal of Evolutionary Biology 2001 14 406 417 10.1046/j.1420-9101.2001.00286.x

[B45] Tsoar H Blumberg DG Wenkart R Breckle S-W, Yair A, Veste M Formation and geomorphology of the north-western Negev sand dunes Arid Dune Ecosystems - The Nizzana Sands in the Negev Desert 2008 Berlin: Springer-Verlag 25 48 full_text

[B46] Wright S Evolution and the Genetics of Populations Variability within and among Natural Populations 1978 4 Chicago: University of Chicago Press 406 417

[B47] Randi E Baker AJ Mitochondrial DNA Molecular Methods in Ecology 2000 Malden, MA: Blackwell Science 136 167

[B48] Matson CW Baker RJ DNA sequence variation in the mitochondrial control region of red-backed voles (Clethrionomys) Molecular Biology and Evolution 2001 18 1494 1501 11470840 10.1093/oxfordjournals.molbev.a003935

[B49] Hingston M Goodman SM Ganzhorn JU Sommer S Reconstruction of the colonization of southern Madagascar by introduced Rattus rattus Journal of Biogeography 2005 32 1549 1559 10.1111/j.1365-2699.2005.01311.x

[B50] Stewart JB Freyer C Elson JL Wredenberg A Cansu Z Trifunovic A Larsson N Strong purifying selection in transmission of mammalian mitochondrial DNA PLoS Biology 2008 6 e10 10.1371/journal.pbio.0060010 18232733 PMC2214808

[B51] Haythornthwaite AS Dickman CR Long-distance movements by a small carnivorous marsupial: how Sminthopsis youngsoni (Marsupialia: Dasyuridae) uses habitat in an Australian sandridge desert Journal of Zoology 2006 270 543 549 10.1111/j.1469-7998.2006.00186.x

[B52] Goring-Morris AN Goldberg P Late quaternary dune incursions in the southern Levant: archaeology, chronology and palaeoenvironments Quaternary International 1990 5 115 137 10.1016/1040-6182(90)90031-X

[B53] Tchernov E Yom-Tov Y, Tchernov E The paleobiogeographical history of the southern Levant The zoogeography of Israel 1988 Dordrecht, The Netherlands: Dr. W. Junk Publishers 159 250

[B54] Brand S Abramsky Z Body Masses of Gerbilline Rodents in Sandy Habitats of Israel Journal of Arid Environments 1987 12 247 253

[B55] Wahrman J Gourevitz P Extreme chromosome variability in a colonizing rodent Chromosomes Today 1973 4 399 424

