# Supplementary material for: Conservation genetics of a rare Gerbil species: a comparison of the population genetic structures and demographic histories of the locally rare Pygmy Gerbil and the common Anderson's Gerbil
Source: BMC Ecol. 2010 Jun 2;10:15. doi: 10.1186/1472-6785-10-15 (PMC2887812; doi:10.1186/1472-6785-10-15)
Supplement: Additional file 1 — Pairwise PhiPT values for G. henleyi's CR & CO2 sequences. The low values indicate low levels of differentiation between populations. Probability values based on 999 permutations are above the diagonal and PhiPT values are below the diagonal. Note that Mamshit (indicated in italics) is the only location from the inner sands. [file 1472-6785-10-15-S1.PDF]

Pairwise PhiPT values for *G. henleyi*'s CR sequence

	Agur South	Agur North	Mifrasit	Mashabim	Mamshit
Agur South	0.000	0.373	0.299	0.328	0.410
Agur North	0.000	0.000	0.352	0.327	0.251
Mifrasit	0.014	0.000	0.000	0.301	0.118
Mashabim	0.000	0.000	0.000	0.000	0.262
Mamshit	0.000	0.037	0.144	0.063	0.000

Pairwise PhiPT values for *G. henleyi*'s CO2 sequence

	Agur South	Agur North	Mifrasit	Mashabim	Mamshit
Agur South	0.000	0.408	0.434	0.247	0.303
Agur North	0.000	0.000	0.440	0.145	0.411
Mifrasit	0.000	0.000	0.000	0.237	0.429
Mashabim	0.030	0.033	0.021	0.000	0.299
Mamshit	0.000	0.000	0.000	0.000	0.000
