# Supplementary material for: Conservation genetics of a rare Gerbil species: a comparison of the population genetic structures and demographic histories of the locally rare Pygmy Gerbil and the common Anderson's Gerbil
Source: BMC Ecol. 2010 Jun 2;10:15. doi: 10.1186/1472-6785-10-15 (PMC2887812; doi:10.1186/1472-6785-10-15)

Gerbillus andersoni allenbyi CO2

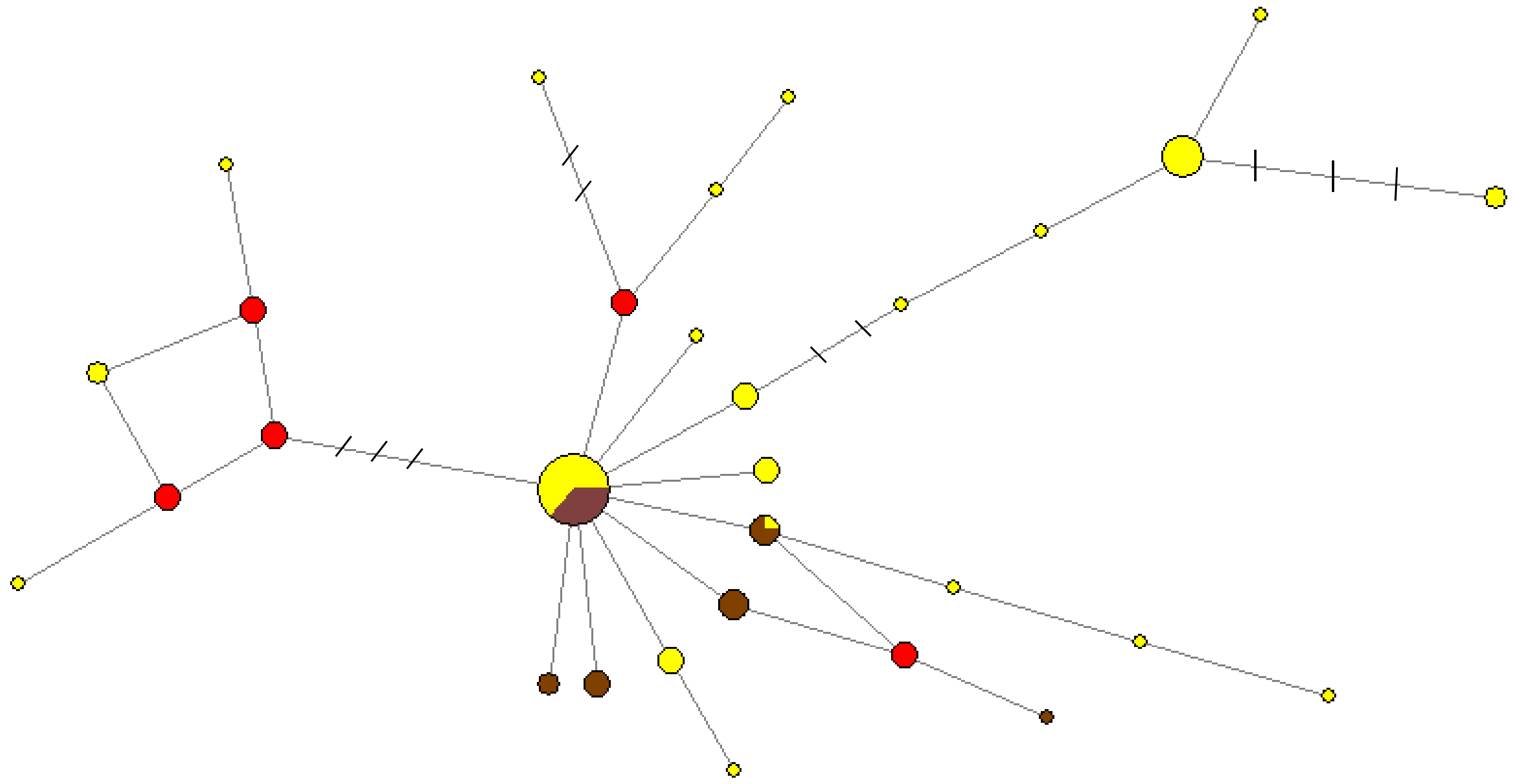

Gerbillus henleyi CO2

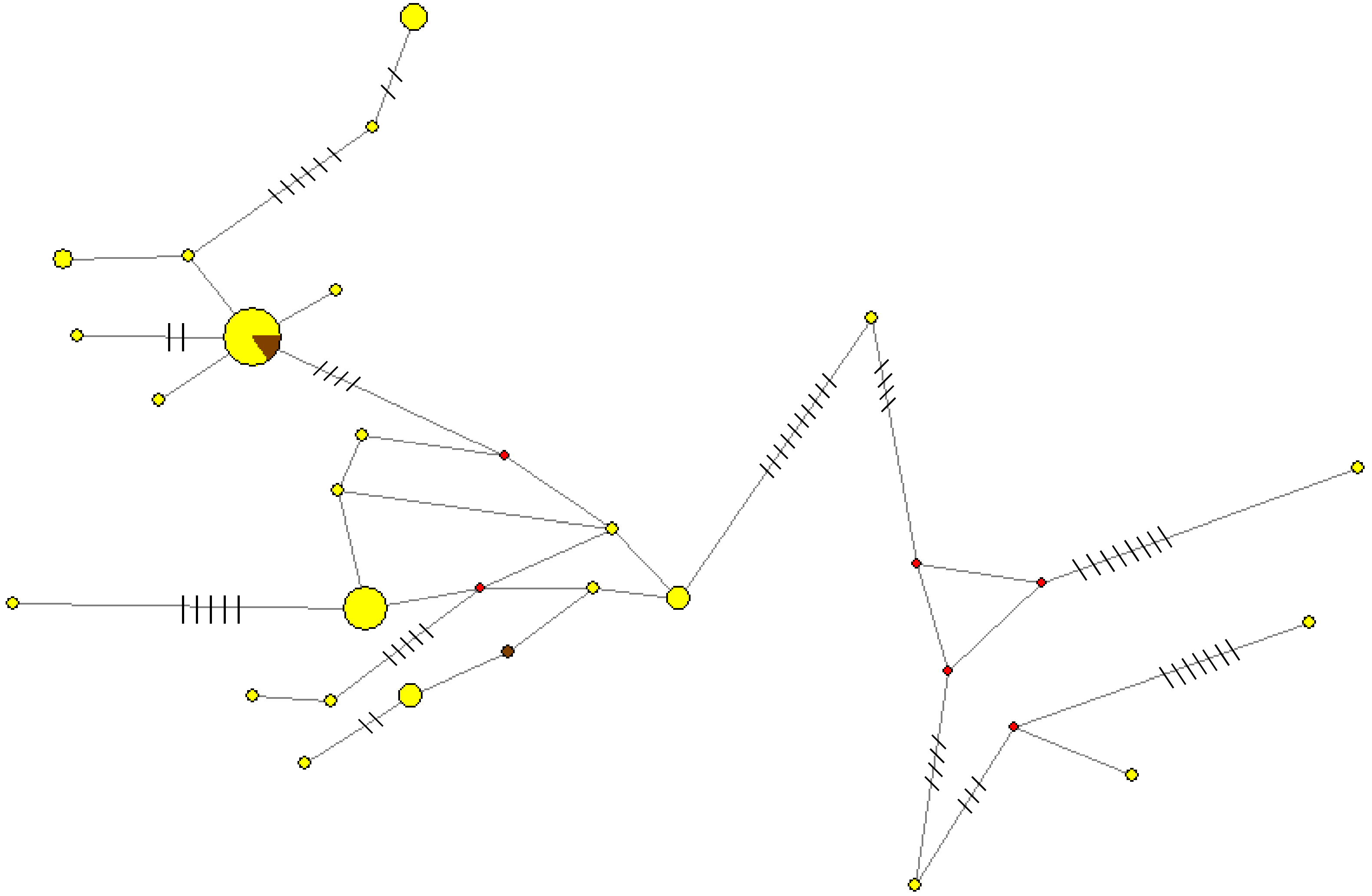

Gerbillus henleyi CR

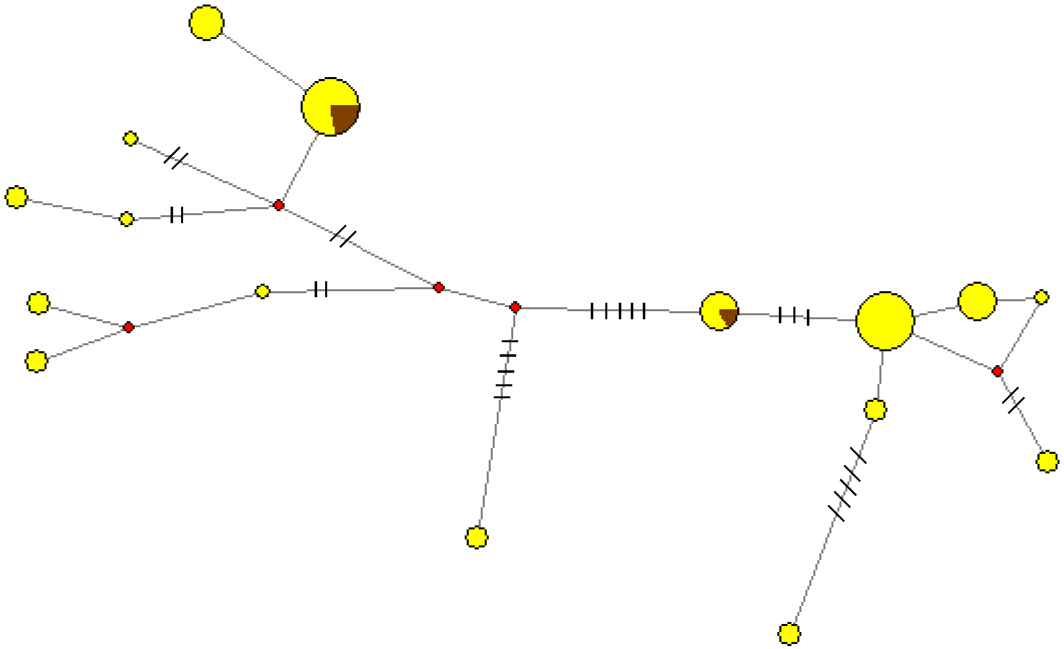

Gerbillus henleyi CR+CO2

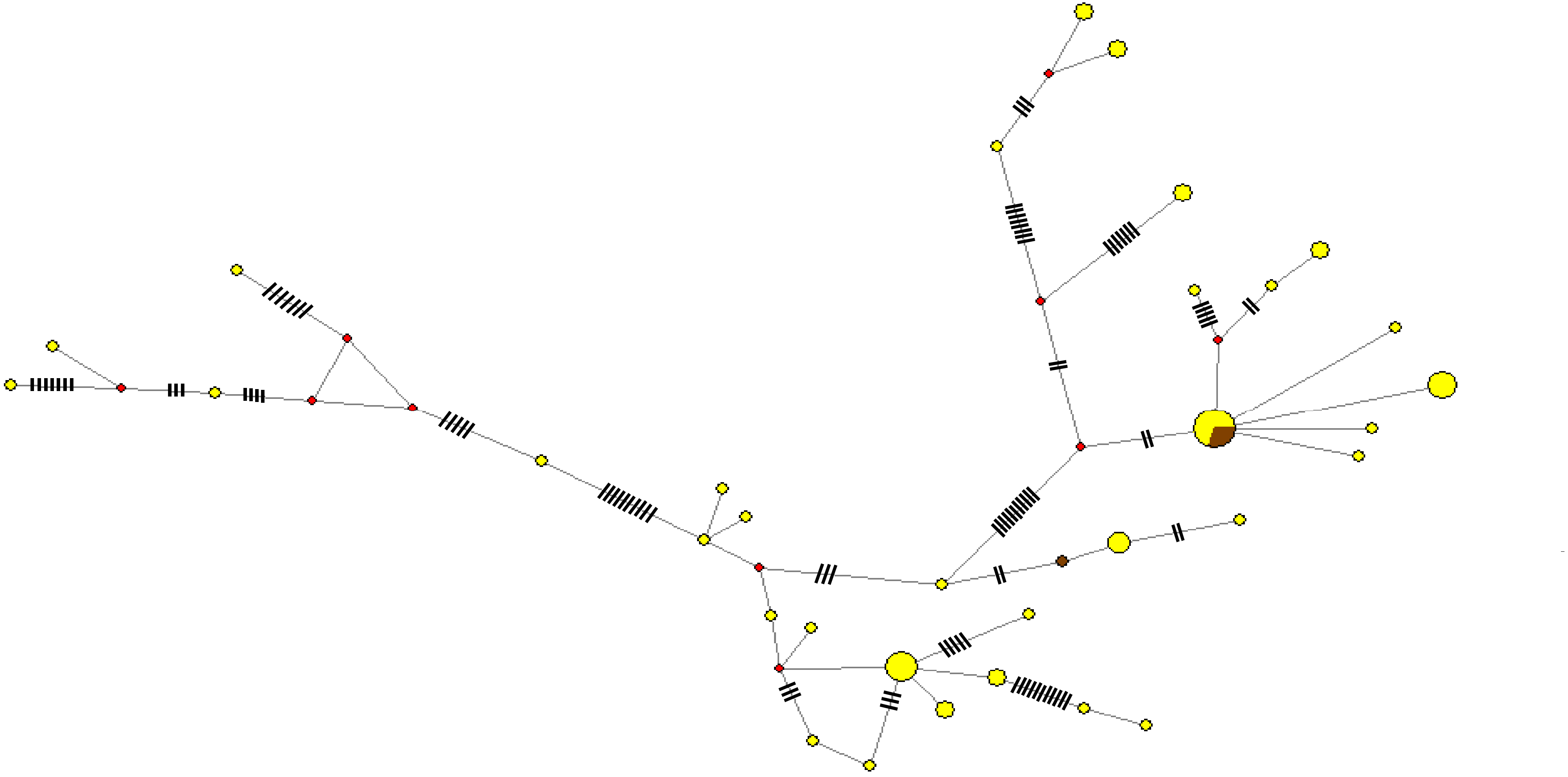

Supplement: Additional file 2 — Haplotype networks of the four sequences (G. a. allenbyi CO2, G. henleyi CO2, CR and concatenated sequence). The networks describe the distances between haplotypes for the three sequences. Circle sizes are proportional to the number of individuals with a certain haplotype. Yellow areas represent individuals from the western Negev sands; brown areas represent individuals from the inner sands. Red circles represent theoretical intermediate haplotypes. The number of mutations is represented by ticks on the lines; a clean line indicates one mutation. In the G. a. allenbyi network, there appear haplotypes which are unique to the inner sands, and are clustered close to a common haplotype which is shared by both regions. In the G. henleyi CR network, there appear no haplotypes which are unique to the inner sands, and no clustering is evident. In the CO2 and concatenated sequence network, one haplotype is unique to the inner sands, but it shows no clear connection to the other inner sands haplotypes. [file 1472-6785-10-15-S2.PDF]
