# Supplementary figures and images for: Conservation genetics of a rare Gerbil species: a comparison of the population genetic structures and demographic histories of the locally rare Pygmy Gerbil and the common Anderson's Gerbil
Source: BMC Ecol. 2010 Jun 2;10:15. doi: 10.1186/1472-6785-10-15 (PMC2887812; doi:10.1186/1472-6785-10-15)

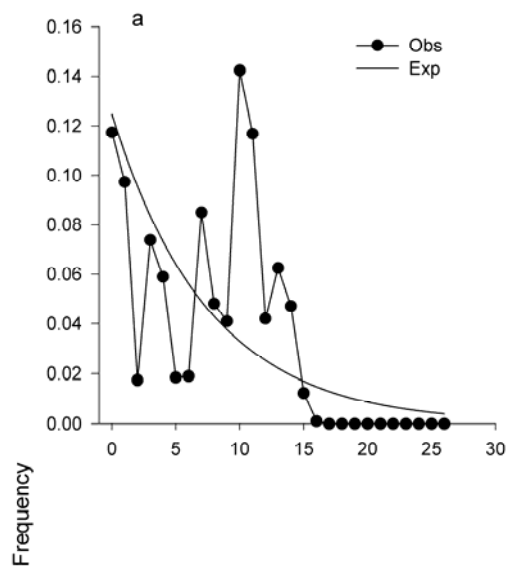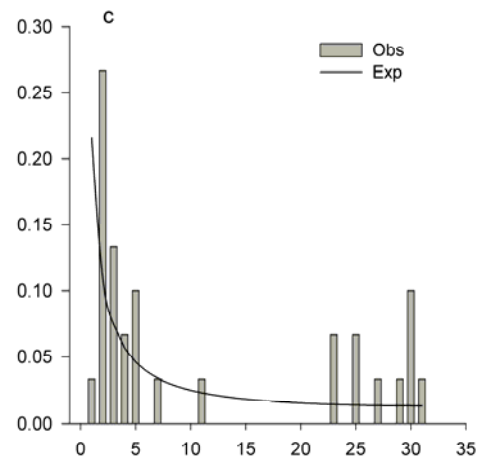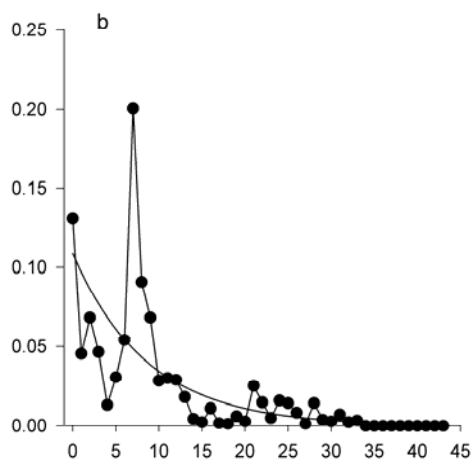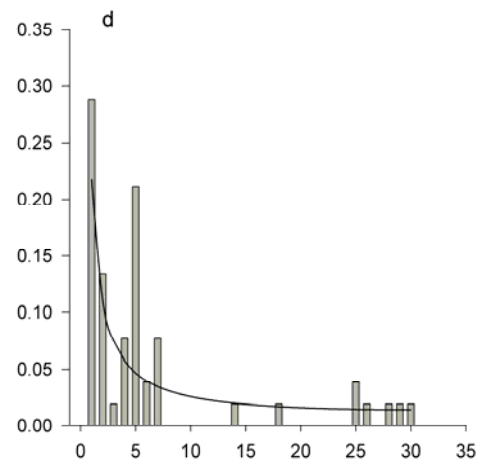

Supplement: Additional file 3 — Pairwise differences and allelic frequency graphs for G. henleyi's CR (a, c) and CO2 (b, d). The ragged pattern in the pairwise differences graphs for both sequences in G. henleyi indicates that this species has maintained a stable population size over a long period of time. The allelic frequency graphs indicate that older mutations (mutations that are shared by many individuals) predominate in G. henleyi, suggesting that this species has experienced a bottleneck. [file 1472-6785-10-15-S3.PDF]
